# Sexual Health Literacy, a Strategy for the Challenges of Sexual Life of Infertile Women: A Qualitative Study

**DOI:** 10.31661/gmj.v9i0.1862

**Published:** 2020-12-28

**Authors:** Zahra Rakhshaee, Raziyeh Maasoumi, Saharnaz Nedjat, Zohreh Khakbazan

**Affiliations:** ^1^Department of Reproductive Health, School of Nursing and Midwifery, Tehran University of Medical Sciences, Tehran, Iran; ^2^Department of Nursing & Midwifery, Rasht Branch, Islamic Azad University, Rasht, Iran; ^3^Nursing and Midwifery Care Research Center, School of Nursing and Midwifery, Tehran University of Medical Sciences, Tehran, Iran; ^4^Epidemiology and Biostatistics Department, School of Public Health, Tehran University of Medical Sciences, Tehran, Iran

**Keywords:** Sexual health, Health Literacy, Infertility

## Abstract

**Background:**

Sexual health literacy enables an understanding and application of sexual health information and has benefits beyond health. Health literacy is an important element for achieving cognitive skills in health promotion. One of the most important problems in sexual health and sexual function in women is infertility. This study aims to explore the dimensions of sexual health literacy among women with infertility.

**Materials and Methods:**

In this qualitative study, a total of 18 individual interviews with 15 Iranian women with infertility, and three key informants, were conducted in infertility centers in Rasht (the North of Iran). Data were collected through in-depth semi-structured interviews using interview guide questions. Data were analyzed using the conventional content analysis approach.

**Results:**

Five themes emerged: informational needs of sexual health, information seeking, informational perception, validation of information, and information application. Sexual issues are taboo in Iranian culture. They are not taught in health and educational centers. All the participants believed that there was a lack of information about the sexual response cycle, preventing sexually transmitted infections, targeted intercourse, and consequences of infertility in sexual life. Participants mentioned the embarrassment, privacy, and lack of centers for sexual health as information-seeking barriers. Searching different sources and questioning the informants were ways for understanding information. Women evaluated the accuracy of the information by considering the validity of the source of information, comparing information from different sources, and asking the experts. They applied the information received about sexual health, satisfaction, and especially targeted intercourse to increase the chance of pregnancy.

**Conclusion:**

Sexual health literacy in infertile women includes different dimensions. It can help promote sexual health, satisfaction, and increasing the chance of pregnancy.

## Introduction

Health literacy is an important element in women’s ability to achieve cognitive skills and their involvement in health promotion and preventive behavior [1]. The World Health Organization (WHO) states that sexual health literacy provides the ability to understand sexual health information and application of that information, decreasing the risk of sexually transmitted infections (STIs), and also providing various benefits beyond health [2]. Sexual health literacy is influenced by gender, age, sexual education, sexual experience, birthplace, and religious affiliation [3]. Svensson *et al*. (2016) stated that sexual health literacy in women opened the door to new understandings of sexual-reproductive health and rights and their engagement in these issues [4]. Many sexual problems are caused by unawareness and wrong beliefs about sexual relationships [5], whereas educating sexual skills is associated with improving sexual function [6,7]. Health literacy could be effective in sexual satisfaction. It affects the attitude of couples towards sexual and marital relationships [8]. In a way, inadequate health literacy can lead to dissatisfaction in couple’s relationship and problems in their marital life; therefore, promoting sexual health literacy is an effective step toward improving marital satisfaction and quality of life [9]. One of the determinative problems in sexual health and function, especially in women, is infertility [10]. Infertility is defined as an inability to get pregnant one year after having regular intercourse without the use of contraceptive methods [11]. According to WHO, about 5-10% of couples worldwide have infertility [12]. Infertility has the characteristics of a crisis and can result in a widespread crisis in the lives of infertile couples [13]; physical and emotional involvements, difficult tests, medical interventions, and scheduled sexual intercourse disrupts women’s sexual function and transforms a couple’s relationship into something mechanical [14,15]. Infertility and its treatment can not only cause sexual dysfunction and decrease the quality of sexual life and intimacy, but it can also become a source for separation and divorce [16-18]. WHO has proposed sexual relationships as a source of psychological and social support in infertility and has emphasized raising the quality of sexual relationships [19]. The study by Saheb-al-Zamani *et al*. (2018) showed that the level of health literacy among Iranian infertile couples was marginal and that the decreased level of health literacy had adverse effects on a couple’s sexual function and satisfaction [8]. Therefore, considering the effect of infertility on the cycle of sexual response [20] and the role of health literacy on sexual function and satisfaction [10], promoting the sexual health literacy of women with infertility is important.

 An extensive literature review showed no studies have yet been conducted about sexual health literacy in infertile women. Also, sexual health literacy is affected by culture, age, target group characteristics, and the context. Therefore, it could not be just evaluated by quantitative studies. Hence, this qualitative study aims to explore the dimensions of sexual health literacy and the experiences of infertile women in Iran.

## Materials and Methods

 The present study was a qualitative investigation with a conventional content analysis approach and the first part of a mixed-method study exploring sexual health literacy among infertile women.

###  Participants and Settings 

 In this study, sampling was purposive and with maximum variety, considering factors such as age, education, employment status, socioeconomic status, cause of infertility, type of infertility, and duration of infertility. The inclusion criteria were Iranian women with primary and secondary infertility (as diagnosis by a specialist physician), willingness to participate in the study, ability to understand, speak, and communicate in the interview in the Persian language. Two specialized infertility centers in the city of Rasht (in the North of Iran), including Mehr Infertility Institute (a private center) and Al-Zahra Infertility Clinic (an educational-governmental center), were selected. A total of 18 individual interviews were conducted from July 2018 to February 2019, of which 15 included women with infertility and three involved key informants, including a gynecologist and two counseling midwives of the infertility centers.

###  Data Collection 

In the present study, deep semi-structured interviews of women with infertility and key informants were used to collect data. At the beginning of each interview, via a checklist, demographic information and participants’ infertility status were obtained. The development of the interview guide was structured around Sørensen’s conceptual model of health literacy [21], to explore knowledge and experiences of sexual health information. Interviews contained some open and total questions and were started with the question of “What do you know about sex and sexual health? What information are you looking for?”. For more clarity, each question was followed with more investigative interview questions, such as asking for examples or more explanation. The duration of each interview varied from 40 to 80 minutes. This sampling procedure continued until data saturation so that no new data were obtained from the interviews.

###  Data Analysis

After gaining permission from the participants, interviews were recorded. In the shortest possible time, after listening several times, interviews were written and typed verbatim. Data were analyzed using conventional content analysis. At first, meaning units were determined, then these units were summarized, and codes were generated. Codes were compared based on their similarities, differences, and categorized. Finally, categories, subcategories, and themes emerged [22].

###  Trustworthiness

To ensure the trustworthiness of the data, Guba and Lincoln criteria, including credibility, dependability, transferability, and conformability, were used [23]. Credibility was determined through long-term involvement with the data, selecting the participants with maximum variety and different experiences, and providing some of the coded texts to the participants for evaluating the accuracy of the researcher’s perception.

 To increase the dependability of the data, the texts of the interviews and the results of the coding and extracted categories were evaluated by two experts in the field of qualitative research, who expressed a high degree of agreement regarding the extracted results. To transferability, the entire research process and actions were written clearly and accurately so that it would be possible for others to follow the research path and the characteristics of the population studied. Two expert qualitative researchers and two external supervisors evaluated some of the interviews, codes, and extracted categories for conformability.

###  Ethical Considerations

 The ethical approval (IR.TUMS.FNM.REC.1397.066) was received from the Committee of Faculty of Nursing and Midwifery of Tehran University of Medical Sciences, and permission to enter infertility centers. Before each interview, the researcher explained the aims of the study, confidentiality, volunteering participation, and the possibility to withdraw from the study at any stage. Informed consent was obtained from all the participants. Interviews were conducted in a private room in the infertility center.

## Results

The results of this study were obtained by analyzing data collected from 18 individual interviews, including 15 interviews with infertile women and three with key informants (two midwives and one gynecologist). The infertile women ranged from 27 to 44 years old with a mean age of 33.06 years. Of the 15 women with infertility, 10 had primary infertility, and 5 had secondary infertility. The duration of marriage was 2 to 20 years with a mean of 6.7 years. The duration of infertility was 1 to 7 years with a mean of 3.6 years. Table-1 shows the characteristics of the participants.

 The main domains of sexual health literacy were extracted into five themes of informational needs of sexual health, information seeking, informational perception, validation of information, and application of information (Table-2).

###  1. Informational Needs of Sexual Health

 This theme contains three categories of recognizing stages of sexual interaction, knowledge of sexual skill as a guarantee for sexual satisfaction, and challenges of sexual life in infertility. Sexual issues are taboo in Iranian culture. They are not taught in health and educational centers. Therefore, all the participants mentioned the need for information about sexual health.

####  1.1. Recognizing of Stages of Sexual Interaction

 Women emphasized the necessity to know male and female genital organs and provide information about the sexual response cycle. The participants raised many points about sexual desires and their effective factors, how to begin sex, signs of sexual stimulation in the body, and orgasm.

*P7: “Sexual relationship education, knowledge of women’s genitalia is really necessary… I really didn’t know; I had never heard about orgasm. I thought that sexual pleasure was just for men.*”

Most women mentioned the need for information about sexual health, methods of transferring and preventing genital infections and sexually transmitted diseases such as gonorrhea, genital warts, genital herpes, AIDS, and hepatitis.

*P11: “We do not know about many of the sexually transmitted diseases. For example, how they would be transmitted and, can we be safe using condoms?.*”

####  1.2. Knowledge of Sexual Skill as A Guarantee for Sexual Satisfaction

 According to the results obtained, knowledge of sexual skills was one of the informational needs for achieving sexual satisfaction. Women emphasized the need for information about methods of gaining sexual skills, including sexual discourse for recognizing sexual interests and desires, fulfilling sexual expectations, and paying attention to each other’s sexual satisfaction. Infertile women mentioned sexual dissatisfaction as a factor for misconduct, incompatibility, and disloyalty in marital life.

*P3: “Sometimes a husband and wife would feel ashamed to tell each other about their sexual desires and fantasies... Good sex would strengthen the marital life, would bring the husband and wife closer to each other … If the wife does not care enough, the man might go for extramarital relationships.*”

####  1.3. Challenges of Sexual Life in Infertility 

 Women stated that their sexual life had been affected by their infertility. They referred to targeted sexual intercourse, focus on getting pregnant during intercourse and required information about the time of ovulation, fertility window period, the position of intercourse, and frequency of intercourse for increasing the chance of pregnancy.

*P4: “How and when you should have an intercourse to increase the chance of pregnancy?... Does a woman’s sexual pleasure have any effect on fertility or not?.*”

Women mentioned the effect of infertility on their sexual function and a decrease in their sexual desire due to having scheduled intercourse, mental business, fear, etc. They considered infertility as a factor threatening their marital life and a potentiating factor for divorce. They also emphasized the provision of information about infertility outcomes in sexual interactions and an appropriate manner for dealing with it.

*P8: “When I consume the drugs, I get colder about sex. I’m not enthusiastic about it. I don’t know whether it is the effect of the drugs or my mind is entangled, or I’m tired.*”

###  2. Sexual Health Information Seeking 

 This theme consists of two categories, including effective factors in information seeking and sources of information seeking.

####  2.1. Effective Factors in Information Seeking

 Due to a lack of official sex education in Iran, all the women interviewed felt the need for information on this subject. Ease of access to information, the ability to search for information, and interest in information were the effective factors in information seeking. They said that the ability to search for information from the internet with mobile phones had accelerated and facilitated access to information. They mentioned that sexual education is a taboo subject, feeling ashamed, being private, and lack of educational health centers for sexual health were barriers to seeking information. As a result, these barriers lead to complacency and a lack of desire for the treatment of sexual problems in couples.

*P5: “Our country’s culture somehow makes us feel ashamed of talking about our sexual issues… It is taboo in society, and nobody teaches us anything.*”

####  2.2. Sources of Information Seeking

 To find information, women searched the internet and published sources as non-human sources, and asked relatives, friends, and healthcare providers, as human sources. For most of them, internet sources and searching public websites (Google) were the main sources of information, and mobile internet was their most accessible source. Since sexual issues are not spoken in the public media of Iran, such as radio and television, books were known as valid sources of information for most women. In most cases, relatives and friends were the most important source for gaining information, and some mentioned their husbands as to the first source of sexual information.

*P15: “I read some of the information about sexual relationships in books, some from the internet… I only talk about these issues to my sister whom I feel comfortable with.*”

Some women mentioned obtaining brief information about contraceptive methods and sexually transmitted infections from midwives in pre-marriage classes. They also mentioned obtaining brief information about their sexual problems and the timing for intercourse from the doctor. In the present study, health care providers were the last source of information for women, as they felt ashamed of asking sexual questions in routine evaluations in the health centers of Iran.

###  3. Informational Perception

 This theme has two categories of information, perception strategy and factors influencing perception and understanding.

####  3.1. Information Perception Strategy

 Searching in various internet websites, using new keywords and online dictionaries for Latin and medical terms, and asking questions from friends, expert acquaintances, and health care providers were strategies employed to better understand and perceive the information.

*P6: “If I read something on the internet and did not understand it, I would search it again on the internet, and if I had any questions, I would ask or quickly search it on the net using my mobile phone.*”

####  3.2. Factors Influencing Perception and Understanding 

 Women mentioned reinforcing and inhibiting factors as the influencing factors of understanding and perceiving sexual health information. Using simple language without Latin and medical terms, classification of the information, using images, and providing information through counseling were reinforcing factors, whereas low educational level, brief and quick provision of information, and information irrelevant to the individual’s needs were inhibiting factors.

*P1: “It was explained using pictures… with a simple language without any scientific and medical terms which I could easily understand.*”

###  4. Validation of Information

 This theme has two categories, separation of valid from invalid sources and assurance of information accuracy.

####  4.1. Separation of Sources

 Women did not trust some of the information that was received from the internet, pornographic movies, experiences of acquaintances, and inexpert individuals; they mostly trusted information received from physicians, midwives, and books. They also considered the information received from scientific and specialized websites as valid.

*P14: “About marital relationships, I’ve heard everyone talking about their experiences that my husband might not like… or the movies that people watch are not correct at all.*”

####  4.2. Assurance of the Information’s Accuracy

 Most women accepted information based on the evaluation. They compared information from different sources, compared them with their own experiences and previous knowledge; more importantly, they evaluated the correctness of the information by asking a physician, midwife, or an expert person.

*P9: “It is better that we ask a physician about the correctness of the information.*”

###  5. Application of the Information

 This theme has two categories of targeted application of the information for getting pregnant and the application of the information for sexual health and satisfaction.

####  5.1. Use of the Information for Getting Pregnant

 All infertile women paid attention to targeted intercourse, such as increasing the number of intercourses around the time of ovulation, the correct manner of intercourse, and resting after intercourse to increase the chance of pregnancy.

*P4: “I know that fertility is a cycle, and the 12th to 18th days are the ovulation days. I have intercourse during these days… I also rest a little after intercourse.*”

####  5.2. Use of the Information for Sexual Health and Satisfaction

 Women applied the information received about good sexual interactions and sexual health. In this regard, they mentioned talking to husbands about sexual desires, having sexual intercourse with calm and enthusiasm, paying attention to cleanliness and adornment, flirting to achieve mental and physical preparation, and paying attention to genital hygiene to prevent infections and sexually transmitted diseases.

*P8: “We start our intercourse by hugging and endearment. I think it is better to start intercourse with flirting.*”

## Discussion

According to the results, in infertile women, sexual health literacy was an individual skill in obtaining sexual health informational needs (including recognition of stages of sexual interaction, targeted intercourse, preventing sexually transmitted infections, recognizing sexual skills, sexual discourse, sexual interests and expectations) through information seeking, informational perception, validation, and active application of information. Therefore, they could promote sexual health, increase the chance of pregnancy, improve interactions, and form a clearer understanding of the responsibilities in sexual relationships, sexual satisfaction, and strengthened marital life. Sexual health literacy is a skill set that incorporates health knowledge and behavior [24]. Information and knowledge about sexual health lead to the formation of attitudes, beliefs, and values [25]. The study of Svensson *et al*. (2016) assessed the sexual health literacy achieved by the women through their descriptions of knowledge gains and shifts in attitudes. The study indicated that women found themselves better informed and more confident in using the understanding gained after receiving sexual health information [4]. In a way, higher levels of sexual health literacy are associated with better sexual performance and higher sexual satisfaction [8], and lower levels of sexual health literacy lead to a decreased rate of condom use and increased possibility of high-risk sexual relationships [26]. In the present study, all the women emphasized the necessity of having sexual health information, including knowledge about the genital organs and sexual response cycle, to improve sexual intercourse. In the study of Bostani *et al*. (2015), most women also did not have the correct information about their genital organs [27]. Unawareness about the anatomy and function of the reproductive system raises many questions in mind [28] and could be one of the reasons for sexual dissatisfaction [29]. The study of Bokaei *et al*. (2017) showed that providing counseling for infertile couples about the cycle of the sexual response and sexual health could impact infertile women’s sexual men [20]. Women considered familiarity with sexual skills as an informational need to start desirable intercourse for achieving sexual satisfaction. Awareness about these issues would help couples have more rational and responsible sexual relationships and have better sexual intercourse [30]. Women believed that their infertility led to focusing on pregnancy and decreased sexual desire. They emphasized that information about the role of infertility in sexual interactions is essential in forming the appropriate response to this malady. In this regard, other studies also showed that infertility and its related treatment would lead to changes in the sexual function, so sexual relationships would be turned into a method for reproduction and pregnancy [31,32].

In the present study, women tried searching for information due to lack of or insufficient information about sexual health. Results from other studies indicated that in Iran, accessibility to scientific and accurate information about sexual health is difficult due to lack of official education, lack of education by parents, low level of knowledge of healthcare providers [33], and embarrassment [34]. Accessibility to information is the first most significant dimension of sexual health literacy [35]. In this study, the most important sources for obtaining information were the internet, books, acquaintances, and friends. Using various sources was in line with the results of Mercer *et al*. (2014) [36]. Embarrassment at having to ask questions about sexual issues and lack of routine evaluation of sexual health resulted in healthcare providers being the last source of information for the women. In this regard, Svensson *et al*. (2016) reported that embarrassment and taboos related to sexual health issues gave rise to knowledge gaps and misconceptions about sexual health [4].

A woman’s ability to fulfill her informational needs depends on her access to various information sources and appreciating the related information [37]. In this study, to appreciate the obtained information, participants searched various sources and asked questions from the informants. They mentioned reinforcing factors, such as using images and simple language without medical terms and inhibiting factors such as low educational level or inappropriateness of the information as effective factors in perceiving the information. In line with the present study results, other studies also showed that health information would be more easily understood through simple language, audio and video equipment, numbers, statistics, and proportionate information [38-40]. Validation of the information was another dimension of sexual health literacy. The women evaluated the accuracy of the obtained information in several ways; by comparing the information from different information with each other, comparing new information with that obtained from specialists (e.g., physicians, midwives), and comparing the obtained information their previous knowledge and experiences. According to results from other studies, comparing the information obtained from different sources was a method for judging the information [41,42], and the similarity of information in various websites would increase trust in that information [43,44].

To arrive at appropriate decisions about their health, individuals should be able to understand and apply the information [45]. Participants stated that after receiving information about sexual health and understanding and grasping this information, they tried to apply it to improve their sexual health, increase their chance of pregnancy, and increase their sexual satisfaction. It means empowerment of the individual and increasing understanding about sexual issues and relationships. In other words, in addition to the new knowledge acquired, women feel that the information changed their lives [4]. Therefore, having a desirable level of sexual health literacy would increase the individual’s skill in analyzing, judging, discourse, decision-making, and changing sexual behavior and would empower them to provide, maintain, and promote their sexual health [46,47].

## Limitations

 Some women were reluctant to be interviewed due to the privacy of the sexual issue, shame, and embarrassment. Therefore, participants were assured of the confidentiality of information, and interviews were conducted in a private room. Also, sampling was performed only in Rasht city, but an attempt was made to ensure the study embraced maximum diversity in age and education.

## Conclusion

 By recognizing different dimensions of sexual health literacy in women with infertility, specialists of sexual health will be able to provide proportionate sexual health information for patients with infertility. Therefore, besides promoting sexual health, improvement of sexual interactions and gaining sexual satisfaction would help strengthen their marital life. It could also have a positive effect on one of the main goals of infertile couples, pregnancy.

## Acknowledgment

 This article was a part of a Ph.D. thesis (IR.TUMS.FNM.REC.1397.066) in reproductive health that was approved by the Tehran University of Medical Sciences. The authors would like to thank the research deputy of the nursing and midwifery faculty of Tehran University of Medical Sciences, the manager of the Mehr Infertility and Medical Institute of Rasht, Dr. Mehr Afza, Research deputy of Gilan University of Medical Sciences and participants for their sincere cooperation.

## Conflict of Interest

 The authors report no conflicts of interest.

**Table 1:** A Summary of the Participants’ Demographic Characteristics

**Characteristics of the infertile women (n=15)**						
**No.** **participant**	**Age** **(years)**	**Education level**	**Duration of** **marriage** **(years)**	**Duration of** **infertility** **(years)**	**Type** ** of** ** infertility**	**Cause** **of** **infertility**
**P1**	28	Associate	8	3	Primary	Female
**P2**	42	Bachelor	20	3	Secondary	Male
**P3**	36	Bachelor	3	3	Primary	Female- male
**P4**	33	Associate	11	3	Secondary	Unknown cause
**P5**	41	Graduate	3	2	Primary	Male
**P6**	31	Graduate	3	2	Secondary	Female- male
**P7**	28	Bachelor	6	4	Primary	Unknown cause
**P8**	30	Graduate	2	1	Primary	Female- male
**P9**	29	Graduate	5	5	Primary	Female
**P10**	32	Bachelor	3	3	Secondary	Unknown cause
**P11**	27	Graduate	4	2	Primary	Unknown cause
**P12**	44	Graduate	10	5	Secondary	Unknown cause
**P13**	29	High school diploma	6	6	Primary	Male
**P14**	35	Graduate	7	7	Primary	Male
**P15**	31	High school diploma	10	6	Primary	Female
**Characteristics of the Key informants (n=3)**						
**No.** **participant**	**Age** **(years)**	**Education level**	**Education**	**Work experience (years)**	**Clinics**
**P16**	37	Graduate	Midwifery	11	Private
**P17**	36	Graduate	Midwifery	10	Public
**P18**	50	PhD	specialist Gynecologist &infertility	22	Private

**Table 2:** Classification of Theme, Categories and Subcategories Regarding Sexual Health in Infertile Women

**Theme**	**Categories**	**Subcategories**
**Informational needs of sexual health**	Recognizing of stages of sexual interaction	To know male and female genital organs Sexual response cycle Sexual health, preventing genital infections and STIs
	Knowledge of sexual skill as a guarantee for sexual satisfaction	Sexual discourse Fulfilling sexual expectations Paying attention to each other’s sexual satisfaction
	Challenges of sexual life in infertility	Targeted sexual intercourse Consequences of infertility in sexual life
**Sexual health information seeking**	Effective factors in information seeking	Facilitators Barriers
	Sources of information seeking	Non-human sources Human sources
**Informational perception**	Information perception strategy	Searching different sources Questioning the informants
	Factors influencing perception and understanding	Reinforcing factors Inhibitors factors
**Validation of information**	Separation of sources	Valid sources Invalid sources
	Assurance of the information accuracy	Comparing information Asking from expert
**Application of the information**	Use of the information for getting pregnant	Timing for Intercourse The correct manner of intercourse
	Use of the information for sexual health and satisfaction	Mental and physical preparation for sexual relationship Genital hygiene
